# Supplementary material for: Feasibility and agreement between TTE and intracavitary ECG for PICC tip positioning in adult oncology patients: A single-centre exploratory study
Source: PLoS One. 2026 Apr 2;21(4):e0345105. doi: 10.1371/journal.pone.0345105 (PMC13046106; doi:10.1371/journal.pone.0345105)
Supplement: S1 Data — An anonymized dataset supporting the findings of this study. (ZIP) [file pone.0345105.s001.zip › Supp/S4_Protocol.docx]

Accuracy study of trans-thoracic wall cardiac ultrasound (TTE) guided real-time central venous catheter tip localization

Subject Source: Nanjing Drum Tower Hospital

Subject number:

Person-in-Charge：Kai Xin

Department: Department of Oncology

Contact Person: Mian Zhou

Research start time: 2022.04 to 2024.12

Version number: V1.0

Version date: 2023.03.14

**Produced by Gulou Hospital of Nanjing University School of Medicine**

**Programs Programs Abstract Summary**

| Project name | Accuracy study of trans-thoracic wall cardiac ultrasound (TTE) guided real-time localization of central venous catheter tip |
| --- | --- |
| Purpose of the study | a. To establish the technique of real-time positioning of catheter tip by cardiac ultrasound and optimize the operation procedure;  b. Conduct clinical studies to further investigate the consistency of trans-thoracic wall cardiac ultrasound localization of central venous catheter tip position and postoperative CT. |
| Study design | Diagnostic study |
| Total number of cases | 30 cases |
| Case selection | *Inclusion Criteria*  *Malignant tumors proposed for cyclic infusion of chemotherapeutic agents requiring placement of PICC, PORT catheter;*  *Patients aged >18 years old;*  *Agree to participate in this study and sign the informed consent for this study.* |
|  | *Exclusion criteria*  *Patients who cannot tolerate ultrasound monitoring after recent major chest surgery;*  *Patients who cannot tolerate CT examination;*  *Patients with contraindications to central venous catheter placement ;*  *Suffering from thrombophilia;*  *Those with abnormal coagulation function.* |
| Treatment Options | Those who signed the informed consent and met the inclusion and exclusion criteria were enrolled.  Patients requiring central venous catheterization  Undergoing TTE-guided catheterization |
| Evaluation of efficacy | *Indicators of effectiveness evaluation (primary and secondary outcome indicators)*   1. *Primary outcome indicators:*   *Precision of catheter tip localization (accuracy)*   1. *Secondary outcome indicators:*   *Surgical time, economy* |
|  | *Safety evaluation indicators*  *Incidence of surgical site infection, incidence of intraoperative adverse events, catheter ectasia* |
| Statistical methods | SPSS 20.0 statistical software was used to perform t-test, xo2 test and Fisher's exact test to analyze the safety, efficacy and economy. |
| Study period | 2023.04 to 2024.12 |

Table of Contents

[I. Background of the study 5](#_Toc110801521)

[Purpose of the Study 7](#_Toc110801522)

[III. Types of Study Design, Principles and Experimental Steps 7](#_Toc110801523)

[Case selection 9](#_Toc110801524)

[V. Research Methods and Technical Route 9](#_Toc110801525)

[Observation items and testing time point 12](#_Toc110801526)

[VII. Efficacy assessment criteria 12](#_Toc110801527)

[Observation of adverse events 13](#_Toc110801528)

[IX. Quality control and quality assurance of the study 15](#_Toc110801529)

[X. Data safety monitoring 15](#_Toc110801530)

[XI. Statistical Processing 16](#_Toc110801531)

[XII. Ethics of Clinical Research 17](#_Toc110801532)

[XIII. Collection and Storage of Samples/Specimens 17](#_Toc110801533)

[XIV. Study Progress 18](#_Toc110801534)

[XV. References 18](#_Toc110801535)

[Pre-test data recording 21](#_Toc110801536)

List of Abbreviations

| English Abbreviations | Full name in English | Chinese Full Abbreviation |
| --- | --- | --- |
| TTE | Transthoracic echocardiography | Transthoracic echocardiography |
|  |  |  |
|  |  |  |
|  |  |  |
|  |  |  |
|  |  |  |

# Background

The central venous catheter is the first line catheter for chemotherapy administration and parenteral nutrition ^([) (1) (~2) (])^; the tip position of the central venous catheter is very important, and the diagnosis of catheter tip ectasia after the placement of the catheter implies the need for tip repositioning, which will increase the risk of infections, radiological injuries, and greater healthcare expenditures ^([) (3) (])^. According to the INS infusion guidelines ^([) (4) (])^, the central venous catheter tip should be optimally positioned in the lower 1/3 of the superior vena cava at or near the CAJ, and catheter tip ectasia can result in varying degrees of complications.

With the development of technology, new catheter localization techniques are emerging, and the localization techniques that are widely used in the clinic include in vitro measurement, imaging localization, and intracavitary electrocardiographic localization ^([) (5])^.

In vitro measurement is to measure the length from the pre-positioning site to the third intercostal space, of which the most traditional method is to measure the length from the puncture point to the right sternoclavicular joint, and then the length from the right sternoclavicular joint downward reflexed to the third intercostal space, in the patient's prone position, with the arm positioned at 90° to the body ^([) (6) (])^. Later on, some scholars have continuously improved the extracorporeal measurement method, such as the one-line measurement method, the elbow transverse measurement method, the caliper (formula) measurement method and so on ^([) (7) (])^, but the variability of these measurements is large, which is not enough to guide clinical practice, and some researchers believe that the extracorporeal measurement method is greatly affected by the patient's height, arm length, and the width of the rib cage, and therefore can only be used as a preliminary prediction of the depth of placement of the catheter^([) (8) (]).^ .

Imaging localization methods include pre-catheterization chest radiography and post-catheterization chest radiography. The pre-tube placement method ^([) (9) (])^refers to the preoperative chest X-ray, the distance from the right sternoclavicular joint to the sixth thoracic vertebra is measured on the chest radiograph, and the distance from the puncture point to the right sternoclavicular joint is added to the distance from the puncture point to the right sternoclavicular joint when the patient is in the lying position with the arm at 90° to the body, and the sum of these two distances is the length of the tube placement. Post-catheterization chest radiography is considered the "gold standard" for positioning the tip of the central venous catheter ^([) (5) (])^. X-ray chest radiographs are taken after the completion of catheterization, and professionals determine whether the tip of the catheter is ectopic or not through the chest radiographs, and if it is ectopic, the position of the catheter tip is adjusted according to the ectopic condition and the judgment of professionals.

Intraluminal electrocardiogram localization method is a hot spot of research at home and abroad at present. This method is based on the theory of electrical activity of the heart and relies on the judgment of changes in electrical signals within the lumen to locate the catheter tip in the central vein ^([) (10])^. Studies have shown that the intracavitary ECG-guided catheter tip localization is more precise than in vitro measurements and X-ray localization techniques ^([) (11) (~12) (])^. Moreover, the endocavity ECG method can be operated at the bedside, real-time localization is achieved, there is no radiological injury, and it can be applied to pregnant women, children, and neonates, and it reduces the incidence of related complications^([) (1) (3) (~1) (~1) (4) (])^.

In addition, there are ultrasound localization method, electromagnetic navigation localization technology, central venous pressure localization method. Among them, ultrasound localization method can realize real-time localization at the bedside and is easy to operate, with less damage to patients. According to the research of domestic scholars, the sensitivity of trans-thoracic wall echocardiography (TTE) in catheter tip localization is as high as 98.6% ^([) (1) (5) (])^, but ultrasound interpretation relies on ultrasound-qualified professionals, and the technology lacks the corresponding technical specifications. Electromagnetic navigation localization technique ^([) (1) (6) (~1) (7) (])^ is currently more widely used in foreign countries, This method borrows sensors to sense the magnetic field generated by the guidewire in the catheter, and the operator can observe the position of the guidewire through the monitor, which is also a kind of real-time localization technique, has a high localization accuracy rate^([) (1)(8) (])^, but fewer studies have been carried out in China on the related technology. Central venous pressure localization method ^([) (1) (9) (])^ is to use the principle that the central venous pressure of the superior vena cava vein is 5~12cmH_2_O. After delivering the central venous catheter, connecting the tee tube and measuring the central venous pressure (CVP), if the CVP< is 12cmH_2_O, it proves that the tip of the catheter arrives at the superior vena cava vein.

In clinical practice, our team has found that although there are various techniques for catheter tip positioning, there are still cases of difficult and imprecise positioning. Combined with this, our team has used transthoracic cardiac ultrasound (TTE) to assist catheter tip positioning in the process of catheter placement, which has achieved good results.

TTE can make up for the shortcomings of chest X-ray, which is unable to locate the catheter tip in real time and has radioactive damage. Although chest X-ray has been considered the "gold standard" for central venous catheter tip localization ^([) (20) (])^, it also has certain shortcomings. Firstly, in terms of the accuracy of localization, chest X-ray cannot directly show the exact position of the CAJ, and it is necessary to indirectly determine the position of the tip of the catheter through the anatomical marking of the image, and under the influence of the patient's respiratory movement, the position of the tip of the catheter will move up and down along with respiratory movement, which reduces the accuracy of the localization of the chest X-ray ^([) (4) (])^. Second, chest X-rays have a certain amount of radioactive damage to the body, while TTE does not have such damage. Finally, chest radiographs need to be taken in the imaging department, so it is difficult to quickly transfer the patient to take chest radiographs to explore the catheter position if there is difficulty in placing the catheter during the placement process; in addition, after the end of the placement, if chest radiographs are taken to show that the tip of the catheter is poorly positioned, it is necessary to adjust the position of the catheter tip for a second time, which increases the risk of infection and the cost of treatment.

TTE can be used in patients with unstable waveforms on intraluminal ECG, insignificant P waves or no P waves. Intraluminal ECG positioning techniques are affected by individual differences, and there is no conclusive evidence regarding the relationship between changes in intraluminal ECG P-wave morphology and catheter tip position, and there are difficulties in eliciting characteristic P waves ^([) (21])^. In addition, according to the 2021 edition of the INS Infusion Therapy Guidelines, intracavitary ECG is prohibited for use in patients with no P waves or abnormal cardiac rhythms, whereas TTE compensates for the shortcomings of intracavitary ECG localization at this point.

Based on the previous clinical practice, i.e. TTE was used to investigate the catheter tip position when the catheterization nurse had difficulty in locating the catheter, the research team accumulated the corresponding clinical experience and kept the data for analysis, and the following findings were made: i. TTE locates the catheter tip position visually. ii. Usually, the patient performs cardiac ultrasonography in the left lateral recumbent position. Due to the limitation of the catheterization position, we tried to observe the patient in the lying position from different views (short-axis view of the great arteries, four-chambered cardiac view, and subxiphoid bi-atrial view), and we found that the visibility of the three views was 60%, 80%, and 10%, respectively, and therefore we took the four-chambered cardiac view as the standard view for observation; second, the selection of the time point for observation. We found that when the catheter was determined to be in the right atrium, the position of its tip was changing with the cardiac cycle, and it was necessary to select the time point as a standard. After statistical analysis, we found that the length of the catheter into the right atrium was most accurately estimated based on the distance from the tip of the catheter to the interventricular septum at the end of the atrial systole period, VL2 (see Appendix for study data).

# Purpose of the study

1. primary purpose: to establish real-time cardiac ultrasound localization technique and to establish a standardized operating procedure (SOP) for this technique

2. secondary purpose: to conduct a clinical study to further validate the accuracy of trans-thoracic wall cardiac ultrasound localization of the catheter tip.

# III. Types of study design, principles and experimental steps

1. Study design

1. Type of study design: Diagnostic study
2. Research center: Nanjing Gulou Hospital Jiangbei Branch

2. Indications

Patients with malignant tumors requiring central venous catheterization for cyclic infusion of chemotherapy drugs; patients >18 years old; patients who agree to participate in the study and sign the informed consent form.

3. Endpoints

Patients' post-catheterization CT images.

4. Procedure

Patients sign an informed consent form

Screening

Failure

Withdrawal

Success

TTE is used to localize the catheter tip during catheterization.

Primary efficacy measures

Secondary efficacy measures

Safety evaluation

End of trial

Detection of catheter tip position by CT after catheterization

#

# Case selection

1. Number of cases

It was planned to enroll 30 subjects in our Comprehensive Oncology Center for this study.

1. Inclusion Criteria
2. Those with malignant tumors who are proposed to undergo cyclic infusion of chemotherapeutic agents requiring placement of PICC, PORT catheter;
3. Patients aged >18 years;
4. Agreed to participate in this study and signed the informed consent for this study.
5. Exclusion criteria
6. Patients who cannot tolerate ultrasound monitoring after recent major chest surgery;
7. Patients who cannot tolerate CT examination;
8. Patients with contraindications to central venous catheter placement;
9. Patients with thrombophilia;
10. Those with abnormal coagulation function.
11. Exclusion Criteria

Patients who did not use TTE to locate the catheter tip after enrollment.

1. Criteria for termination of the trial

Patients with intolerance during catheterization were unable to complete the trial.

# V. Research Methods and Technical Route

1. Subject enrollment

Patients were enrolled in the order of catheterization.

1. Intervention/Study drug name and specification

In this study, trans-thoracic wall cardiac ultrasound was used to locate the position of the catheter tip, all intraoperative ultrasound exploration steps were done by one doctor, tube placement steps were done by one nurse, and postoperative CT examination was performed in the imaging department of our hospital.

(1) Instruments

Ultrasonic instrument

(2) Methods

Ultrasound localization catheter tip operation procedure:

a. Patient position preparation: the patient takes a flat lying position, and the catheterization arm is placed at 90° to the torso;

b. Disinfecting and spreading the towel: measure the arm circumference, disinfect the catheter placement site, and spread the towel;

c. Placement of catheter: the catheterization nurse punctures the needle under ultrasound guidance and places the catheter;

d. Cardiac ultrasound-assisted catheter tip localization: ultrasound probe to explore the four-chambered heart section, observe the imaging effect (if the observation effect is not good, observe the short-axis section of the great arteries and subxiphoid section in turn), ultrasound doctor and the placement nurse to cooperate with the placement nurse to send the catheter until the doctor can observe the tip of the catheter under the ultrasound, retain the image to calculate the distance between the tip of the catheter and the septum at the end of cardiac contraction period, L, and the placement nurse to pull out the catheter to the outside. The catheterization nurse pulls the catheter outward for the length of L.

e. Proper fixation of the catheter: The placement nurse continues to complete the subsequent steps to properly fix the catheter.

3. Research intervention program

After patients signed the informed consent form, those who met the inclusion and exclusion criteria were enrolled;

Subjects received TTE-guided catheter placement, and the catheter tip position and procedure time were recorded during placement;

At the end of catheter placement, subjects had CT taken;

The patients were followed up postoperatively for the occurrence of complications (infections);

Analyzing whether there is consistency between TTE-guided catheter tip positioning and CT.

| Personnel Assignment | Assignment |
| --- | --- |
| One physician | Responsible for the ultrasound localization part of the catheter placement process. |
| One catheterization nurse | Responsible for the catheterization process, cooperating with the doctor to complete the tip positioning. |
| One recorder | A recorder is responsible for recording data on site. |

4. Combination of drugs

This study does not involve the combination of drugs.

5. Research Flowchart

| Trial Flow Chart | | | |
| --- | --- | --- | --- |
| Window period  Trial Procedure | Visit 1  Screening period | Visit 2  Trial period | Visit 3  Observation Period |
|  | -1~0 days | 1 day | 2~ 3 days |
| Signed informed consent | × |  |  |
| Demographic information | × |  |  |
| Medical history and treatment history | × |  |  |
| Determination of inclusion/exclusion criteria | × |  |  |
| Randomization | × |  |  |
| Body temperature | × | × | × |
| Catheter placement |  | × |  |
| Observation and documentation of catheter tip position |  | × |  |
| CT examination |  |  | × |
| Complications | × | × | × |
| Adverse events |  | Continuous observation | |
| Surgical time |  | × |  |
| Nurse Confidence Index |  | × |  |
| Study Summary |  | × | |

6. Study-related laboratory tests

Blood tests (RBC/WBC/PLT/HBG) and coagulation tests (PT/APTT/FIB/D-Dimer/INR) will be completed during the screening period. During the trial and observation periods, if patients develop high fever and are reasonably suspected of having catheter-associated infections, blood cultures are required to confirm the diagnosis of catheter-associated infections.

7. Study-related imaging

Postoperative chest CT should be performed to confirm the location of the catheter tip under imaging.

# VI. Observation items and testing time point

| Observations | Time point of testing |
| --- | --- |
| Precision of catheter tip position | Day of surgery |
| Length of surgery | Day of surgery |
| Confidence of the catheterization nurse | Day of surgery |
| Postoperative Infection | Postoperative day 3 |
| Incidence of intraoperative adverse events | Day of surgery |

# VII. Efficacy Assessment Criteria

1. Effectiveness index
2. Main observation indexes
3. Precision of catheter tip position (Time: the day of surgery)
   1. DEFINITION: The American Society of Infusion Nursing considers the optimal location for the tip of the central venous catheter to be the superior vena cava junction (CAJ), i.e., the junction of the superior vena cava with the right atrium or the lower third of the superior vena cava (SVC) in its vicinity. In this study, the catheter tip was considered to be accurately positioned when it was shown on postoperative CT to be in the CAJ or the lower third of the SVC.
   2. Calculation: $导管尖端位置精准度=\frac{导管尖端位置精准病例数}{总样本量}\times100\%$
   3. Evaluation method: chest CT was taken immediately after the subject's operation and interpreted by a physician with relevant experience.
4. Secondary evaluation indicators
5. Surgical time (time: the day of surgery)
   1. Definition: The length of time from the beginning of the catheterization operation by the catheterization nurse to the fixation of the catheter.
   2. Evaluation method: The length of time was recorded by the recorder.
6. Economy (time: within 3 days after the intervention)
   1. The time spent by the subjects on catheterization, the number of cases and time spent on secondary postoperative adjustment of the catheter tip position, and the medical costs were counted.
   2. Statistical analysis of economic-benefit indicators.
7. Confidence of the catheterization operator
   1. Definition: Confidence of the catheter placement operator in positioning the catheter tip to the optimal position with ultrasound assistance.
   2. Evaluation method: A qualitative interview was conducted in the form of a postoperative interview with the catheterization operator, with the following outline: 1. How long has your catheterization/harboring experience been? 2. Have you had difficulty in positioning the catheter tip in the past? 3. Do you feel more confident in your operating technique when ultrasound-assisted catheterization is performed? 4. Do you feel more confident in positioning the catheter in the optimal position when ultrasound-assisted catheterization is performed? 5. think that the use of ultrasound interferes with your catheterization?
8. Safety Indicators
9. Incidence of surgical site infection (Time: 3 days postoperatively)
   1. Definition: in this study, it refers to infections occurring at the PICC placement site or at the PORT capsular bag, with clinical manifestations including localized redness, swelling, warmth, pain and tenderness.
   2. Calculation:$术后感染发生率=\frac{发生术后感染的病例数}{总样本量}$× 100%


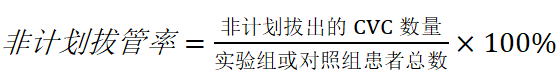


- 1. Evaluation method: the clinician judged whether the infection occurred according to the subject's symptoms and the Chinese Guidelines for the Prevention of Surgical Site Infections.

1. Incidence of intraoperative adverse events (Time: the day of surgery)
   1. Definition: This study refers to adverse events that occurred during tube placement.
   2. Calculation: Intraoperative adverse event incidence rate =$\frac{发生不良事件的病例数}{总样本量}$

# VIII. Observation of adverse events

Adverse event (AE), refers to all adverse medical events that occur after a subject receives a test drug, which can be manifested as symptoms signs, diseases, or abnormalities in laboratory tests, but are not necessarily causally related to the test drug.

Serious Adverse Event (SAE) means an adverse medical event involving death, life-threatening, permanent or serious disability or loss of function, hospitalization or prolonged hospitalization of the subject, congenital anomalies, or birth defects that occurs after the subject receives the investigational drug.

Adverse drug reaction (ADR), any harmful or undesired reaction that occurs in a clinical trial that may be related to the medicinal product being tested. There is at least a reasonable possibility of a causal relationship between the trial medicinal product and the adverse event, i.e. correlation cannot be ruled out.

Suspected and Unexpected Serious Adverse Reaction (SUSAR), a suspected and unanticipated serious adverse reaction where the nature and severity of the clinical presentation exceeds the information available in the investigator's brochure for the test drug product, the specification of the marketed drug product, or the summary of product characteristics.

Subjects will be closely observed by the investigator and all adverse events reported by the subject or observed by the investigator from the time of randomization to the end of the study or early withdrawal from the study must be documented in the medical record or CRF, including laboratory and ancillary investigations, and should include, at a minimum, the name of the AE, the time of onset, time of termination, the severity, the presence or absence of corrective therapy, regression, and causal relationship to the study. causality, etc.

Changes in the subject's vital signs, physical examination, clinical presentation, and laboratory tests should be evaluated during the study. At the time of signing the informed consent form, the name and telephone number of an investigator whom the subject can contact in case of an emergency, or report of any medical symptoms, or occurrence of a subject-related AE must be provided.

Toxic reactions or AEs that existed prior to the subject's participation in the clinical study were recorded as AEs only if the grading was found to have increased by one or more grades from the baseline assessment during the study.

Adverse event description:

INTRAOPERATIVE PAIN: During the tube placement maneuver, the patient was unable to tolerate the pain caused by the ultrasound probe pressing on the chest wall.

SOLUTION: Maintain communication with the patient during the procedure, and if the patient is unable to tolerate the pain, immediately stop the ultrasound probe operation and ask another placement nurse to complete the catheter implantation by using electrocardiographically guided catheter tip positioning (conventional method).

| **CTCAE classification** | **Equivalent** | **Definition** |
| --- | --- | --- |
| Grade 1 | Mild | Mild discomfort that does not affect daily life or functioning |
| Grade 2 | Moderate | Discomfort that interferes with daily life and function, no indication for treatment |
| Grade 3 | Severe | Symptoms, affecting daily life and function, with indication for treatment |
| Grade 4 | Life-threatening/disability/loss of function | Life-threatening, indications for urgent treatment, physically disabled status or mental retardation. |
| Grade 5 | Death | An adverse event that results in death. |

AE and Study Relevance Analysis Table

|  | Definitely | Likely | Likely | Doubtful | Unlikely |
| --- | --- | --- | --- | --- | --- |
| Reasonable chronology with medication/interventions | + | + | + | + | - |
| Known type of medication/reaction | + | + | + | + | -Highly unlikely. |
| Cessation of intervention Symptom reduction or disappearance | + | + | ± | ± | - |
| Response recurs after re-intervention | + | ? | ? | ? | -No explanation in terms of disease, comorbidities |
| Cannot be explained by disease, comorbid medications | + | + | -I'm sorry. | ± | -It's not explained by diseases, comorbidities. |

# Quality control and quality assurance of the study

(1) Adequate communication with patients participating in the trial study so that they understand the risks of the trial and obtain their informed consent and cooperation; (2) All applications of ultrasound in the trial were completed by a doctor with relevant qualifications; (3) The tube placement operation part of the trial was completed by a nurse with relevant qualifications; (4) The case collection form was designed in advance, and the researcher responsible for the collection of data was trained in the relevant knowledge to ensure the accuracy and timeliness of data (4) Design the case collection form in advance and train the researcher in charge of data collection to ensure the accuracy and timeliness of data collection.

# X. Data Safety Monitoring

A data safety monitoring program will be developed according to the risk level of the clinical study. All adverse events will be recorded in detail, appropriately handled and tracked until they are properly resolved or the condition is stabilized, and serious adverse events and unintended events will be reported to the Ethics Committee, the competent department, the sponsor and the drug regulatory authority in a timely manner in accordance with the regulations; the principal investigator will conduct a cumulative review of all adverse events on a regular basis, and convene a meeting of the investigators to assess the risks and benefits of the study when necessary; the double-blind trial will be able to Double-blind trials can be urgently unblinded when necessary to ensure the safety and rights of the subjects; independent data monitors will be arranged for studies with greater than minimal risk to monitor the study data at , and independent data safety monitoring committees will be set up for high-risk studies to monitor the cumulative safety and efficacy data in order to make a recommendation on whether to continue with the study or not.

# XI. Statistical Processing

1. Principles of statistical analysis

The statistical analysis of this trial will be carried out by statistical professionals who will be involved in the entire process from study design, statistical analysis of trial results, and ultimately the development of a statistical analysis plan prior to the locking of the study data in order to complete the statistical analysis report.

The software used for statistical analysis will be SAS version 9.4 or above. Statistical description and testing of data will be based on the characteristics of the data, and applicable descriptors and tests will be selected.

Quantitative data will be described using the number of cases, mean, standard deviation, median, upper quartile, lower quartile, minimum and maximum values; categorical data will be described by the number of cases and percentage of each category.

Comparisons of the general conditions of the two groups will be analyzed using appropriate methods according to the type of indicator; group comparisons of quantitative information will be made using the group t-test (chi-square, normal distribution) or Wilcoxon rank-sum test according to the distribution of the data (if the t-test is not applicable); categorical information will be analyzed using chi-square or exact probability (if the chi-square test is not applicable); and hierarchical information will be analyzed using the rank-sum test.

All statistical tests were two-sided, and a p-value less than or equal to 0.05 would be considered statistically significant for the differences tested (unless otherwise specified).

2. Baseline analysis

Baseline analysis was based on the full analysis set (FAS).

The number of enrolled and completed cases in the two groups at each center was summarized, with different dataset sizes, a list of specific shedding cases, and a comparison of shedding rates between the two groups. Subjects' basic demographic information (age, weight, etc.), medical and medication history were described and compared between the two balanced groups.

3. Effectiveness evaluation indexes

Validity evaluation is based on the Full Analysis Set (FAS) and the Compliance Program Set (PPS).

4. Main evaluation index

The primary evaluation metric is catheter tip localization accuracy.

5. Secondary evaluation indexes

The secondary evaluation indexes of this trial are catheterization time, confidence index of catheterization nurses and economy. In the comparison between the two groups, the t-test or Wilcoxon rank-sum test was used to measure the data according to the distribution of , and the chi-square test or Fisher's exact probability method was used to count the data.

6. Safety evaluation index

Safety evaluation was based on the safety analysis set (SS).

7. Adverse events

Adverse events, serious adverse events, device-related adverse events (or serious adverse events) that occurred during the study were described in terms of number of cases, number of cases, and incidence rate, and the specific manifestations, frequency, severity, and relevance to the device were described for all adverse events. Describe the type, number of cases, and incidence of device defects.

8. Vital signs

Statistical descriptions of changes in vital signs from baseline to post-treatment time points were performed for each group separately, including the number of subjects, mean, standard deviation, median, minimum, and maximum values. Measurement data were analyzed by group t-test or Wilcoxon rank-sum test, and count data were analyzed by two independent samples chi-square test or Fisher's exact probability method.

# XII. Ethics of clinical research

The clinical research will follow the World Medical Assembly Declaration of Helsinki and other relevant regulations. The clinical study will be implemented only after the trial protocol is approved by the Ethics Committee prior to the commencement of the study. Before each subject is enrolled in the study, it is the responsibility of the investigator to provide the subject or his/her agent with a complete and comprehensive description of the purpose, procedures and possible risks of the study, and to sign a written informed consent form, which should let subjects know that they have the right to withdraw from the study at any time, and that the informed consent should be retained as a document of the clinical study for reference. Subjects' privacy and data confidentiality will be protected during the study.

# XIII. Sample/Specimen Collection and Storage

This study does not involve collection and storage of samples/specimens.

# XIV. Progress of the study

April 2023 - July 2023 Completion of methodology establishment, research project, and ethical review;

Jul 2023 - May 2024 Completion of validation of this study;

May 2024-December 2024 Write the paper and popularize the application in the clinic according to the test results.

# XV. List of participants

| Name | Unit | Title/Profession | Assignment | GCP Training (Time) |
| --- | --- | --- | --- | --- |
| Xin Kai | Nanjing Gulou Hospital | Resident/Clinical | Program design/clinical operation | 2020.12 |
| Yuan Ling | Nanjing Gulou Hospital | Chief Nurse/Nursing | Program design guidance | 2022.12 |
| Li Shanping | Nanjing Gulou Hospital | Chief Nurse/Nursing | Quality Control |  |
| Wang Chenjuan | Nanjing Gulou Hospital | Nurse Practitioner/Nursing | Clinical Operations |  |
| Wang Lin | Nanjing Gulou Hospital | Nurse Practitioner/Nursing | Clinical Operation |  |
| Zhong Rong | Nanjing Gulou Hospital | Nurse Practitioner/Nursing | Clinical Operation |  |
| Zhou Coronation | Nanjing University School of Medicine | Graduate Student | Coordinator |  |
| Kang Yubiao | Nanjing University of Traditional Chinese Medicine | Graduate Student | Recorder |  |

# XVI. References

1. D'Souza PC, Kumar S, Kakaria A, Al-Sukaiti R, Al-Baimani K, Hamid RS, Mittal AK, Al-Balushi M, Burney IA, Al-Moundhri MS. Complications and Management of Totally Implantable Central Venous Access Ports in Cancer Patients at a University Hospital in Oman. Sultan Qaboos Univ Med J. 2021 Feb;21(1):e103-e109 . doi: 10.18295/squmj.2021.21.01.014. Epub 2021 Mar 15. PMID: 33777430; PMCID: PMC7968907.
2. Böll B, Schalk E, Buchheidt D, Hasenkamp J, Kiehl M, Kiderlen TR, Kochanek M, Koldehoff M, Kostrewa P, Claßen AY, Mellinghoff SC, Metzner B, Penack O. Ruhnke M, Vehreschild MJGT, Weissinger F, Wolf HH, Karthaus M, Hentrich M. Central venous catheter-related infections in hematology and oncology: 2020 updated guidelines on diagnosis, management, and prevention by the Infectious Diseases Working Party (AGIHO) of the German Society of Hematology and Medical Oncology (DGHO). Medical Oncology (DGHO). Ann Hematol. 2021 Jan;100(1):239-259. doi: 10.1007/s00277-020-04286-x. Epub 2020 Sep 30. PMID: 32997191; PMCID: PMC7782365.
3. Liu YND, He JA, Song YL. Meta-analysis of the effect of intracavitary electrocardiography on PICC tip localization[J]. Nursing Research,2017,31(36):4646-4652. DOI:10.3969/j.issn.1009-6493.2017.36.016.
4. Gorski LA, Hadaway L, Hagle ME, Broadhurst D, Clare S, Kleidon T, Meyer BM, Nickel B, Rowley S, Sharpe E, Alexander M. Infusion Therapy Standards of Practice, 8th Edition. J Infus Nurs. 2021 Jan-Feb 01;44(1S Suppl 1):S1-S224. doi: 10.1097/NAN.0000000000000396. PMID: 33394637.
5. Li KJ. Research progress of PICC catheter tip localization methods[J]. Nursing Research,2020,34(19):3471-3474. DOI:10.12102/j.issn.1009-6493.2020.19.019.
6. CAMP-SORRELL M,CAMP-SORRELL D. Access device guidelines: recommendations for nursing practice and education[M]. 3rd ed. Pittsburgh:Pittsburgh Oncology Nursing Society,2011:170.
7. Jiang FZ,Wu LL,Ye XH,et al. Progress of in vitro measurement of central venous catheter placement length via peripheral vein[J]. PLA Nursing Journal,2017,34(5):40-43,48. DOI:10.3969/j.issn.1008-9993.2017.05.011.
8. ZHANG Xiaoju,HU Yan,LI Quanlei,et al. Systematic evaluation of PICC in vitro measurement methods[J]. Journal of Nursing,2014,29(6):78-82. DOI:10.3870/hlxzz.2014.06.078.
9. WU Yan,WANG Bei,WANG Kaihui. Observation on the effect of extracorporeal measurement by chest radiograph localization method before PICC placement[J]. Journal of Nursing,2016(2):63-64. DOI:10.16460/j.issn1008-9969.2016.02.063.
10. Mauro Pittiruti, Giancarlo Scoppettuolo, Antonio La Greca, Alessandro Emoli, Alberto Brutti, Ivano Migliorini, Laura Dolcetti, Cristina Taraschi. Gennaro De Pascale; The EKG Method for Positioning the Tips of PICCs: Results from Two Preliminary Studies. *Journal of the Association for Vascular Access* 1 December 2008; 13 (4): 179-186. doi: <https://doi.org/10.2309/java.13-4-4>
11. Yin YX, Gao W, Li XY, Lu W, Deng QH, Zhao CY, Liu XR, Zhou C, Hou WB, Lu ST, Liu G, Wang LN, Li MQ, Zhang HJ. Insertion of peripherally inserted central catheters with intracavitary electrocardiography. with intracavitary electrocardiogram guidance: a randomized multicenter study in China. J Vasc Access. 2019 Sep;20(5):524-529. doi: 10.1177/ 1129729818819732. epub 2018 Dec 31. PMID: 30596472; PMCID: PMC6699060.
12. Liu G, Hou W, Zhou C, Yin Y, Lu S, Duan C, Li M, Toft ES, Zhang H. Meta-analysis of intracavitary electrocardiogram guidance for peripherally inserted central catheter placement. J Vasc Access. 2019 Nov;20(6):577-582. doi: 10.1177/1129729819826028. Epub 2019 Mar 6. PMID: 30838913.
13. Xiao AQ, Sun J, Zhu LH, Liao ZY, Shen P, Zhao LL, Latour JM. Effectiveness of intracavitary electrocardiogram-guided peripherally inserted central catheter tip placement in premature infants: a multicentre pre-post intervention study. Eur J Pediatr. 2020 Mar;179(3):439-446. doi: 10.1007/s00431 -019-03524-3. epub 2019 Dec 1. PMID: 31788740.
14. Yu C, Shulan L, Juan W, Ling L, Chun-Mei L. The accuracy and safety of using the electrocardiogram positioning technique in localizing the peripherally inserted central catheter tip position: a systematic review and meta-analysis. Nurs Open. 2022 May;9(3):1556-1563. doi: 10.1002/nop2.932. Epub 2021. Jun 16. PMID: 34132498; PMCID: PMC8994971.
15. Wang J, Lin M, Wei L, et al. A controlled clinical study of two methods of PICC catheter tip localization[J]. Chinese Modern Nursing Miscellaneous Zhi,2018,24(3):336-339. DOI:10.3760/cma.j.issn.1674-2907.2018.03.021.
16. Lelkes V, Kumar A, Shukla PA, Contractor S, Rutan T. Analysis of the Sherlock II tip location system for inserting peripherally inserted central venous catheters. Clin Imaging. 2013 Sep-Oct;37(5):917-21. doi: 10.1016/j.clinimag.2013.04.009. Epub 2013 Jul 15. PMID: 23867159.
17. Takakura M, Fujii T, Suzuki S, Nishiwaki K. Interference of a ventricular assist device with magnetic navigation during insertion of Sherlock 3CG™, a bedside peripherally inserted central catheter. J Artif Organs. 2022 Jun;25(2):105-109. doi: 10.1007/s10047-021-01293-1. Epub 2021 Sep 15. PMID: 34524593. 34524593.
18. Dale M, Higgins A, Carolan-Rees G. Sherlock 3CG(®) Tip Confirmation System for Placement of Peripherally Inserted Central Catheters: a NICE Medical Technology Guidance. Appl Health Econ Health Policy. 2016 Feb;14(1):41-9. doi: 10.1007/s40258-015-0192-3. PMID: 26293389; PMCID: PMC4740556.
19. SUN Yuanyuan,NI Chunxiang,CHEN Bei,et al. Clinical application of central venous pressure waveform to guide tip positioning of high pressure resistant injectable peripherally placed central venous catheters[J]. PLA Nursing Journal,2018,35(11):53-56. DOI:10.3969/j.issn.1008-9993.2018.11.014.
20. Zaghloul N, Watkins L, Choi-Rosen J, Perveen S, Kurepa D. The superiority of point of care ultrasound in localizing central venous line tip position over time. Eur J Pediatr. 2019 Feb;178(2):173-179. doi: 10.1007/s00431-018-3269-9. Epub 2018 Oct 29. PMID: 30374753.
21. Zhou Q, Jiang ZX, Dai YY, et al. Progress of research on the application of characteristic P wave in intracavitary electrocardiogram-guided PICC tip localization[J]. Nursing Research,2020,34(4):641-646. DOI:10.12102/j.issn.1009-6493.2020.04.015.

Pre-test Data Record


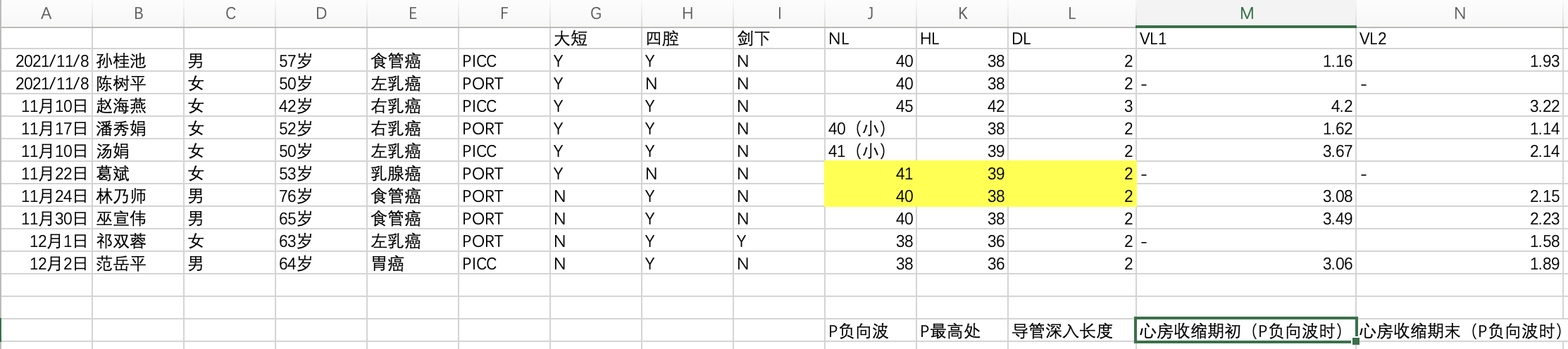


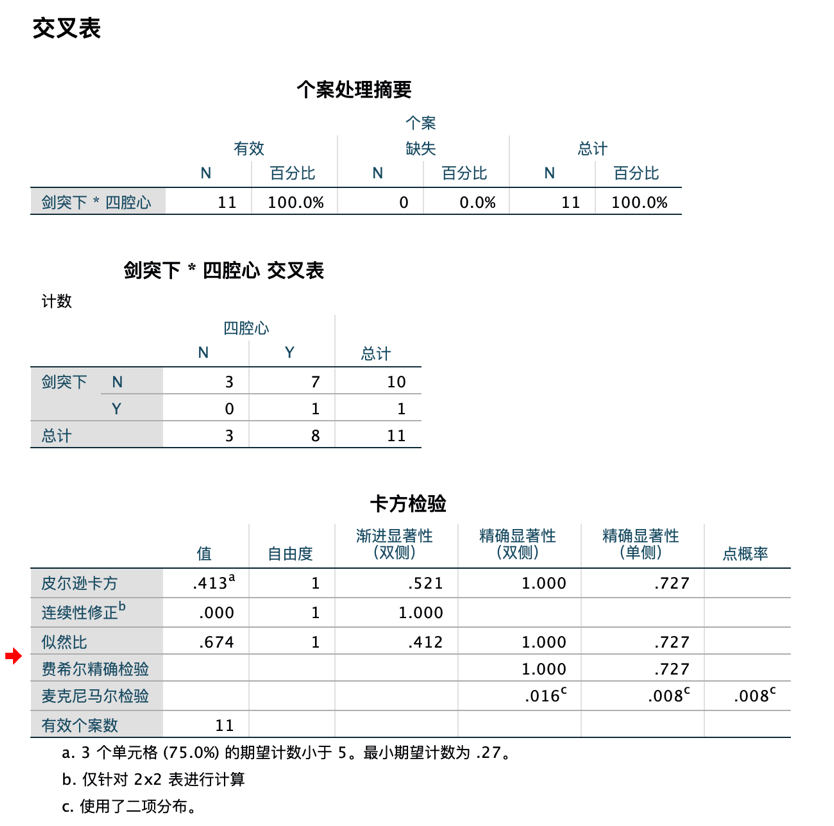


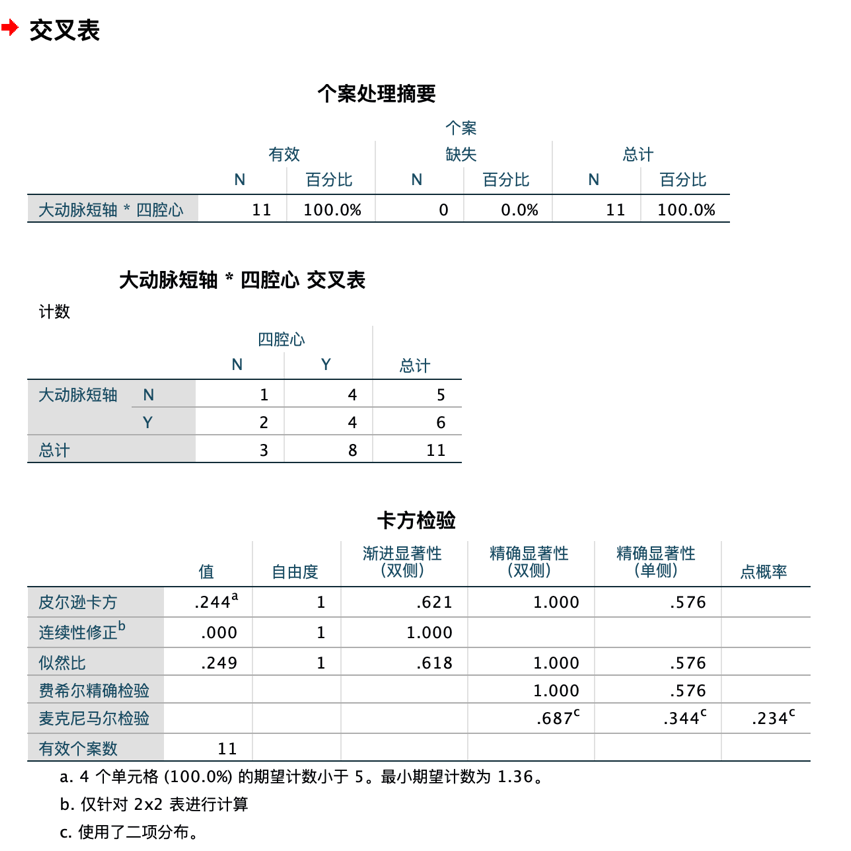


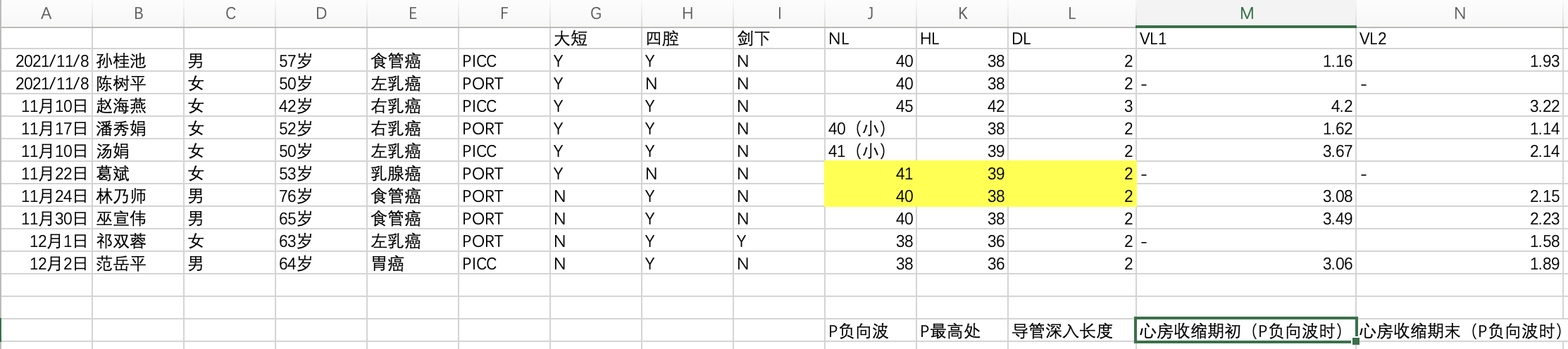


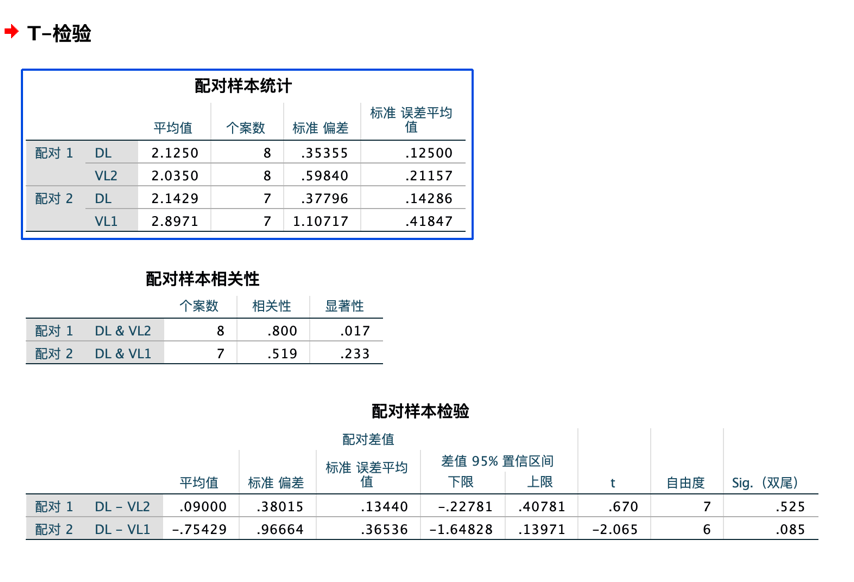


**Investigator statement and protocol signature page**

Investigator Statement:

I agree to comply with the review of the Ethics Committee and to start the execution of the clinical trial after approval, to report promptly to the Ethics Committee any changes in the clinical trial activities and unanticipated problems involving risks to subjects or other persons, and to execute them after reapproval of the ethical review. Comply with the requirements of the Ethics Committee for follow-up review and end-of-study review during the study.

I agree to conduct the clinical trial in strict accordance with the design and specifications of the protocol.

I understand that I may interrupt or terminate this clinical trial at any time if it is necessary to ensure the best interests of the subjects.

I agree that I will personally conduct or supervise this clinical trial and that I will ensure that all investigators in my organization who assist me in conducting this clinical trial are aware of their responsibilities in this clinical trial.

I will strictly adhere to current GCP and the Declaration of Helsinki during the conduct of this clinical trial. I promise that the entire trial will be conducted in a moral, ethical and scientifically sound manner.

During the execution of the clinical trial, I will strictly comply with all laws and regulations related to the clinical trial and protect the rights and interests of patients.

I agree to maintain adequate and accurate medical records and to ensure that these medical records are readily available for audit and inspection in accordance with relevant laws and regulations.

__________________________________ ___________________

Name (in block letters) Signature Date
