# Supplementary material for: Feasibility and agreement between TTE and intracavitary ECG for PICC tip positioning in adult oncology patients: A single-centre exploratory study
Source: PLoS One. 2026 Apr 2;21(4):e0345105. doi: 10.1371/journal.pone.0345105 (PMC13046106; doi:10.1371/journal.pone.0345105)
Supplement: Attachment — Submitted filename: Response to editor and reviewers.docx [file pone.0345105.s003.docx]

**Response to Editor and Reviewers**

October 29, 2025

Editorial Department of PLOS One

Dear editor,

Thanks for giving us an opportunity to revise our manuscript, we appreciate editors and reviewers very much for the positive and constructive comments and suggestions on our manuscript entitled “The accuracy of using the TTE technique in localizing the CVADs tip position: A self-controlled study” (ID: PONE-D-25-30091).

We have studied the comments carefully and have made revision in this paper. Unlike the first version of the manuscript, we have added line numbers in this version. The responses to the comments from reviewers are offered separately, and the corresponding modification position page in the revised manuscript can be found in the letter. Moreover, the revised paragraphs (sentences) are labeled in different colors.

The revised paragraphs based on REVIEWER #1 is labeled in yellow.

The revised paragraphs based on REVIEWER #2 is labeled in gray.

The revised paragraphs based on REVIEWER #3 is labeled in red.

The revised paragraphs based on REVIEWER #4 is labeled in blue.

We would like to express our great appreciation to you and reviewers for comments on our paper. Looking forward to hearing from you.

Thank you and best regards.

Sincerely.

Kai Xin

Reviewer #1:

**The Ethical Approval Document should be in English, but the document presented here is in Chinese and its difficult to understand what's written here.**

**--Response:** Thank you for pointing this out. The Ethics Committee does not provide an English-language version of the certificate, so we must translate it ourselves. The sections in red font within the ethics certificate constitute the translated content. We hope this certificate is acceptable.

**--Change made:** Ethical Approval Document.

**The statistical method should be mentioned below each table.**

**--Response:** We have added the corresponding statistical test descriptions below each table (e.g., t-test, Wilcoxon rank-sum test, Chi-square test).

**--Change made:** Yellow text below the tables.

Reviewer #2:

**The manuscript presents a compelling and well-designed study on the use of transthoracic echocardiography (TTE) for real-time central venous access device (CVAD) tip localization. The study's prospective, self-controlled design is appropriate for the research question, and the use of IC-ECG as a gold standard is a sound methodological choice. The findings that the predicted TTE insertion depth shows good consistency with the IC-ECG method, and that the procedure has a short operation duration and no severe discomfort, strongly support the conclusion that TTE has the potential to be a viable and non-inferior alternative, especially for patients for whom IC-ECG is not suitable. The discussion effectively contextualizes the results, addressing both the strengths and limitations of the TTE method.

Regarding similar works published before, "Ultrasound localization of central vein catheter tip by contrast-enhanced transthoracic ultrasonography: a comparison study with transesophageal echocardiography and chest radiography" by Corradi et al. (2022), investigate,d the use of contrast-enhanced transthoracic echocardiography (CE-TTE) to detect catheter tip misplacements in cardiac surgery patients. The authors of that paper found that CE-TTE, specifically using the epigastric bicaval acoustic view, had high accuracy (97% sensitivity, 90% specificity) compared to the gold standard of transesophageal echocardiography (TEE).

Given these findings from a previous publication, your paper should clearly articulate its novelty. While Corradi et al. focused on CE-TTE in a specific patient population, your study examines non-contrast TTE and compares its accuracy to IC-ECG. Please revise your introduction and discussion to address the following:**

**What specific gap in the literature does your study fill, particularly concerning the use of non-contrast TTE?**

**--Response:** Most prior studies have focused on contrast-enhanced TTE or transesophageal echocardiography (TEE). Evidence regarding the methodological standardization and accuracy of non-contrast TTE for central venous catheter tip localization remains very limited. Our study fills this gap by establishing and preliminarily validating a standardized non-contrast TTE procedure for catheter tip localization.

**How does your validation against the IC-ECG method provide a new or different perspective from Corradi et al.'s comparison with TEE?**

**--Response:** Corradi et al. used TEE as the gold standard, whereas our study employed IC-ECG as the reference. Since IC-ECG is more widely used, convenient, and non-invasive in clinical practice, our design better reflects routine clinical scenarios and directly evaluates the consistency between non-contrast TTE and the conventional clinical standard.

**--Change made:** Page 3, line 108；Page 11, line 326

**Please elaborate on what is new or original in your paper, especially in light of the prior findings that TTE methods can be highly accurate for CVAD tip localization.**

**--Response:** To the best of our knowledge, this study provides one of the first exploratory evaluations of the feasibility of non-contrast TTE compared with IC-ECG in an oncology patient population. Our results suggest that the two techniques show reasonable consistency, with relatively short operation times and acceptable patient discomfort. This approach may represent a potential alternative for patients in whom IC-ECG is not applicable (e.g., those with atrial fibrillation or absent P-waves), and further studies are warranted to confirm its broader clinical value.

**--Change made:** Page 9, line 313

Reviewer #3:

Interesting and very practical paper. Some issues should be added.

**Abstract: please shorten the conclusions.**

**--Response:** We thank the reviewer for this suggestion. We have shortened the conclusion in the Abstract to make it more concise and appropriate for an exploratory study. The revised version avoids overstatement and highlights only the key findings.

**--Change made:** Page 2, line 60

**Do authors think that 13% of mistake is too high?**

**--Response:** We thank the reviewer for this important observation. In our initial analysis, we defined accuracy by taking ECG as the reference standard and considered TTE measurements within ±1 cm as “accurate,” while those exceeding this threshold were classified as “inaccurate,” leading to an error rate of approximately 13%. Upon further reflection, we recognize that this dichotomous definition is not appropriate, since the cavoatrial junction (CAJ) should be regarded as an anatomical region rather than a precise single point. For this reason, we have removed the calculation of “accuracy rate” and “error rate” from the revised manuscript. Instead, we have emphasized the statistical agreement between the two methods, which we believe provides a more valid assessment of methodological consistency and clinical applicability.

**--Change made:** Page 7, line 233

**Methods: it is not clear if authors tested for normality or not**

**--Response:** We thank the reviewer for this helpful comment. In the revised Statistical Analysis section, we have clarified that normality of continuous variables was assessed using the Shapiro–Wilk test. Normally distributed variables were compared with independent-samples t-tests, whereas non-normally distributed variables were analyzed with the Wilcoxon rank-sum test. Categorical variables were analyzed using chi-square or Fisher’s exact tests, as appropriate.

**--Change made:** Page 5, line 190

**Results: the operation time did not differ and this is relevant**

**--Response:** We thank the reviewer for this valuable comment. In the revised Results, we have explicitly reported the comparison of operation times, and in the Discussion, we have highlighted its clinical relevance. Although the mean procedure time for TTE (4.3 minutes) was slightly longer than that of IC-ECG (2.7 minutes), both were within a short and clinically acceptable range. This finding indicates that TTE remains feasible in practice. We also noted in the Discussion that the longer duration for TTE may reflect the operator’s learning curve and the additional imaging steps required.

**--Change made:** Page 10, line 334

**Results baseline features of the patients should be added.**

**--Response:** We thank the reviewer for this valuable suggestion. In the Results, we have already provided detailed baseline characteristics of the study population in Table 1.

**--Change made:** None

**Authors should present features of patients with mistakes, in order to understand the reason of mistake**

**--Response:** We thank the reviewer for this constructive suggestion. In the revised Results, we have added descriptions of the five patients in whom the catheter tip could not be visualized by TTE. These patients commonly exhibited features such as higher BMI, increased chest wall thickness, or a history of chronic obstructive pulmonary disease (COPD), which may have contributed to poor acoustic windows and reduced visualization accuracy.

**--Change made:** Page 6, line 224

Reviewer #4:

**Only 35 patients were enrolled, with 5 excluded due to non-visualization of the catheter tip. This substantially reduces the generalizability of the findings.**

**--Response:** We thank the reviewer for this valuable comment. In the revised manuscript, we have addressed this issue more transparently. In addition to reporting the per-protocol (PP) analysis of 30 patients, we now also present an intention-to-diagnose (ITD) analysis including all 35 patients, in which the five non-visualized cases were classified as “non-diagnostic.” This approach prevents overestimation of TTE accuracy and more realistically reflects its performance in clinical practice. Furthermore, we have emphasized in the Discussion that the small sample size and exclusion of non-visualized cases limit the generalizability of the findings, and that larger, multicenter studies are warranted.

**--Change made:** Page 9, line 301

**IC-ECG was used as the sole gold standard, with no confirmatory radiographic validation.**

**--Response:** We thank the reviewer for this helpful comment. In fact, all patients in our study underwent routine postoperative chest X-ray to confirm catheter position. However, chest radiography has limited precision for catheter tip localization and cannot be regarded as a true gold standard, serving only as a reference. Therefore, IC-ECG was selected as the primary reference standard in our study. In the revised Discussion, we have clarified this point and emphasized that the most accurate confirmatory modalities are transesophageal echocardiography (TEE) or digital subtraction angiography (DSA). Future studies should incorporate these techniques to provide more rigorous validation.

**--Change made:** Page 9, line 294

**The excluded 5 patients represent exactly the cases where TTE is most limited, leading to inflated accuracy estimates.**

**--Response:** We thank the reviewer for this important comment. Indeed, the five excluded cases represent situations in which TTE is most limited, and analyzing only the 30 visualized cases could overestimate accuracy. To address this, in the revised manuscript we have reported both per-protocol (PP) analysis including 30 patients and intention-to-diagnose (ITD) analysis including all 35 patients, in which the five non-visualized cases were classified as “non-diagnostic.” This dual approach provides a more realistic reflection of TTE performance in clinical practice. We also acknowledge this limitation in the Discussion and highlight the need for larger, multicenter studies to validate our findings.

**--Change made:** Page 9, line 301

**The analysis relies on non-significant p-values rather than formal equivalence testing or Bland–Altman agreement assessment.**

--**Response:** We thank the reviewer for this valuable comment. In the revised manuscript, we have added a Bland–Altman analysis to assess agreement between TTE- and IC-ECG–derived insertion depths. Given the exploratory nature and relatively small sample size of our study, we believe that a formal equivalence test (e.g., TOST procedure) would be underpowered and was therefore not performed. We have acknowledged this limitation in the Discussion and highlighted that future large-scale studies should incorporate equivalence testing to more robustly establish clinical equivalence between TTE and IC-ECG.

**--Change made:** Figure 1\2; Page 9, line 301

**All procedures were performed by a single highly experienced operator, which limits external validity.**

**--Response:** We thank the reviewer for this important comment. Indeed, all procedures in this study were performed by a single highly experienced operator. While this ensured procedural consistency, it also limits the external validity of the findings. In the revised Discussion, we have acknowledged this as a limitation and suggested that future studies should involve multiple operators with varying levels of experience to evaluate the reproducibility and generalizability of TTE in broader clinical settings.

**--Change made:** Page 10, line 352

**Pain scores were only recorded for TTE, not for IC-ECG, leading to asymmetry in outcome measurement.**

**--Response:** We thank the reviewer for this thoughtful comment. In this study, pain scores were recorded only for TTE but not for IC-ECG, which introduces asymmetry in outcome measurement. We have clarified this in the revised Methods and Discussion. Because IC-ECG does not involve probe compression or additional manipulation, patients generally experience no pain or discomfort, and pain assessment is therefore not routinely performed in clinical practice. Nevertheless, we acknowledge that this asymmetry may limit the comparability of outcomes. In the Discussion, we have highlighted this limitation and suggested that future studies should apply standardized patient-reported outcome measures to both techniques.

**--Change made:** Page 10, line 352

**Catheter-related complications were monitored only for one week, which is insufficient to assess safety comprehensively.**

**--Response:** We thank the reviewer for this valuable comment. In our study, catheter-related complications were monitored only for one week. This design was chosen because the study was exploratory, single-center, and focused on detecting early postoperative complications such as malposition, bleeding, or infection, which are most likely to occur within the first week. However, we acknowledge that a one-week follow-up is insufficient to capture long-term complications such as thrombosis or delayed infection. In the revised Discussion, we have added this point as a limitation and suggested that future studies should include extended follow-up periods to provide a more comprehensive assessment of safety.

**--Change made:** Page 10, line 352

**--> Please include the five patients in whom the catheter tip could not be visualized in the accuracy analysis, classifying them as “non-diagnostic.”**

**--Response:** We thank the reviewer for this helpful suggestion. In the revised Results, we have included the five previously excluded patients in an intention-to-diagnose (ITD) analysis, classifying them as “non-diagnostic.” The Results now describe both the per-protocol (PP) analysis and the ITD analysis in the text. This approach avoids overestimation of TTE accuracy and provides a more realistic reflection of clinical performance. We have also elaborated on this point in the Discussion to explain its implications.

**--Change made:** Figure 1\2; Page 9, line 301

**- Provide an analysis of predictors of poor visualization (BMI, chest wall thickness, tumor site, prior thoracic surgery).**

**--Response:** We thank the reviewer for this constructive suggestion. In the revised manuscript, we have provided a descriptive analysis of the five patients in whom the catheter tip could not be visualized by TTE. These patients tended to have higher BMI, increased chest wall thickness, or a history of COPD, which may have contributed to poor acoustic windows and limited visualization. Given the small sample size, a formal multivariate analysis was not feasible. Nevertheless, we have added these observations to the Results and discussed them as potential predictors of poor visualization, highlighting the need for larger studies to validate these factors.

**--Change made:** Page 6, line 224

**--> Justify the choice of IC-ECG as the sole gold standard, given its limitations in arrhythmias.
- If feasible, include chest X-ray or fluoroscopic confirmation in at least a subset of patients.**

**--Response:** We thank the reviewer for this important comment. In this study, IC-ECG was chosen as the primary reference standard because it has been extensively validated, is real-time, safe, radiation-free, and widely used in routine clinical practice. We have clarified this rationale in the revised Discussion. We acknowledge that IC-ECG has limitations in patients with atrial fibrillation or absent P-waves; this is precisely why our study emphasizes that TTE may serve as a valuable alternative in such populations. Furthermore, all patients in our study underwent routine postoperative chest X-ray to confirm catheter position. However, chest radiography has limited precision and is not considered a true gold standard, and was therefore used only as supportive information. Because this was a single-center exploratory study, fluoroscopic validation was not feasible; nonetheless, we have added in the Discussion that future research should incorporate more accurate modalities, such as transesophageal echocardiography (TEE) or digital subtraction angiography (DSA), for rigorous validation.

**--Change made:** Page 9, line 294

**--> Apply Bland–Altman analysis to assess agreement between TTE and IC-ECG insertion depths.
- Consider an equivalence test (e.g., TOST procedure) with a clinically meaningful margin (±1 cm).
- Report 95% confidence intervals for accuracy and error rates.**

**--Response:** We thank the reviewer for these valuable suggestions. In the revised manuscript:

1.	Bland–Altman analysis: We have added a Bland–Altman analysis to evaluate the agreement between TTE- and IC-ECG–derived insertion depths.

2.	Equivalence test: Because of the relatively small sample size, a formal equivalence test (e.g., TOST with a ±1 cm margin) would be statistically underpowered and was therefore not performed.

3.	95% confidence intervals: We have already reported 95% confidence intervals for accuracy and error rates in the Results to provide a more transparent and robust interpretation of our findings.

**--Change made:** Figure 1\2; Page 9, line 301

**--> Measure pain scores for both TTE and IC-ECG to allow valid comparison, even if IC-ECG pain is expected to be minimal.**

**--Response:** We appreciate the reviewer’s suggestion. We agree that symmetric patient-reported outcomes would enable a fairer comparison. In our study, pain scores (VAS) were collected only for TTE because, in routine practice, IC-ECG typically involves no probe compression or additional manipulation and is generally painless; therefore, pain scoring was not implemented at the time of data collection. We have clarified this in the Methods and Discussion and listed it as a limitation.

Since the study has concluded and retrospective pain scoring would be biased, we cannot add valid pain data for IC-ECG. As a mitigation, we refrain from making cross-technique claims about pain “superiority,” instead reporting TTE pain distributions descriptively and noting qualitatively that IC-ECG is generally painless.

**--> Discuss the limitation that only one operator was involved.
- If possible, include inter-operator variability data by involving additional sonographers.**

--**Response:** We thank the reviewer for this valuable comment. In this study, all procedures were performed by a single highly experienced operator. While this ensured procedural consistency, it also limits the external validity of our findings. Because this was a single-center exploratory study, we did not include additional sonographers and therefore cannot provide inter-operator variability data. In the revised Discussion, we have acknowledged this limitation and suggested that future studies should involve multiple operators with varying levels of experience to evaluate inter-operator variability and confirm the reproducibility and generalizability of TTE in broader clinical practice.

**--Change made:** Page 10, line 352

**--> Randomize the order of TTE vs. IC-ECG to control for operator learning effect.
- Test TTE performance in patients with atrial fibrillation or absent P-waves, who stand to benefit most.
- Explore use of contrast-enhanced ultrasound (microbubble test) for patients with poor visualization.
- Extend follow-up beyond one week to capture catheter-related complications (malposition, thrombosis, infection).**

--**Response:** We sincerely thank the reviewer for these valuable suggestions:

1.	Randomization of procedure order: In our study, the order of TTE and IC-ECG procedures was not randomized, which may introduce a learning effect bias. We have acknowledged this limitation in the revised Discussion and recommend that future studies adopt randomized procedure order to minimize potential bias.

2.	Patients with atrial fibrillation or absent P-waves: We agree that TTE may be particularly valuable in patients with atrial fibrillation or absent P-waves, who cannot undergo IC-ECG. However, such patients were not included in our study. We have added this as a limitation and emphasized that future studies should specifically evaluate the feasibility and clinical utility of TTE in this population.

3.	Contrast-enhanced ultrasound (microbubble test): We acknowledge that contrast-enhanced ultrasound may improve visualization in patients with poor acoustic windows. As our study was designed to evaluate non-contrast TTE, contrast agents were not applied. We have added to the Discussion that future research should explore contrast-enhanced ultrasound as a supplementary technique in difficult cases.

4.	Extended follow-up: Catheter-related complications were monitored only for one week in this study, focusing on early events. We agree that this duration is insufficient to capture long-term complications such as thrombosis or delayed infection. We have highlighted this as a limitation and recommended extended follow-up in future studies to comprehensively assess safety.

We sincerely thank the reviewers and editor again for their constructive feedback, which has greatly improved our manuscript.
