# Supplementary material for: Feasibility and agreement between TTE and intracavitary ECG for PICC tip positioning in adult oncology patients: A single-centre exploratory study
Source: PLoS One. 2026 Apr 2;21(4):e0345105. doi: 10.1371/journal.pone.0345105 (PMC13046106; doi:10.1371/journal.pone.0345105)
Supplement: Attachment — Submitted filename: Response to Reviewers.docx [file pone.0345105.s004.docx]

Dear Dr. Lionetti,

Dear Reviewers,

We sincerely thank the Academic Editor and all reviewers for their careful evaluation of our manuscript entitled “Feasibility and agreement between TTE and intracavitary ECG for PICC tip positioning in adult oncology patients: a single-centre exploratory study”.

We have carefully considered all comments and have revised the manuscript accordingly. In particular, we have fully adopted the Academic Editor’s recommendations regarding the framing of the study, its limitations, and the interpretation of the findings, in order to ensure methodological transparency and appropriate positioning of this work as an exploratory feasibility study.

Below we provide a point-by-point response

⸻⸻⸻⸻⸻⸻⸻⸻⸻

**Response to Academic Editor**

Comment 1. Please change the title as follows:

“Feasibility and agreement between TTE and intracavitary ECG for PICC tip positioning in adult oncology patients: a single-centre exploratory study”.

Response: We fully agree and have changed the title exactly as suggested by the Academic Editor.

Comment 2. Please rephrase the limitations as follows: “This study has several limitations. It was conducted in a single centre by a single highly experienced operator, which may limit external validity and preclude assessment of operator learning curves. The sample size was relatively small and did not allow for formal equivalence or non-inferiority testing; therefore, the agreement estimates should be interpreted as exploratory. TTE visualization of the catheter tip was not feasible in 5/35 patients (14%), indicating that patient habitus and acoustic windows may restrict applicability in unselected populations. Finally, the cohort consisted solely of oncology patients, and the results may not generalize to other clinical settings.”

Response: We have incorporated this paragraph verbatim into the Limitations section of the revised manuscript.

Comment 3. Please re-phrase the conclusions as follows: “Within these constraints, the present findings suggest that TTE may be feasible for PICC tip confirmation in selected oncology patients, with preliminary agreement between TTE and IC-ECG in cases with successful visualization. However, the non-visualization rate and the single-centre, single-operator design limit generalizability. Larger multicentre studies involving multiple operators and broader patient populations are needed to validate performance and define the clinical role of TTE in this setting.”

Response: We have fully adopted this revised conclusion and replaced the previous version accordingly.

⸻⸻⸻⸻⸻⸻⸻⸻⸻

**Response to Reviewers**

We thank all reviewers for their thoughtful assessment.

Reviewer #1

We thank the reviewer for the positive evaluation and supportive comments. No further changes were requested.

Reviewer #3

We sincerely appreciate the reviewer’s encouraging remarks and recognition of the practical relevance of this study.

Reviewer #5

We thank the reviewer for highlighting the important issues of sample size, statistical power, and the TTE non-visualization rate. These concerns have now been explicitly addressed in the revised manuscript through the expanded Limitations section and the reframed Conclusions, in line with the Academic Editor’s guidance. The study is now clearly positioned as an exploratory feasibility and agreement study rather than a definitive validation, and the results are interpreted accordingly.

We would like to add that we have updated the email addresses of the corresponding authors in the manuscript, and we hope this is acceptable.

We believe that these revisions have substantially strengthened the manuscript by ensuring that the scope, limitations, and interpretation of the findings are fully transparent and appropriately aligned with the exploratory nature of the study. We hope that the revised version now meets the publication criteria of PLOS ONE, and we thank you again for your careful review and guidance.

Sincerely,

Ling Yuan

On behalf of all authors
